# Malaria in the Democratic Republic of Congo: how support programs consolidate elimination gains and protect at-risk populations from recrudescence

**DOI:** 10.1097/MS9.0000000000004085

**Published:** 2025-10-15

**Authors:** Aymar Akilimali, Innocent Mufungizi, Sana Rasheed, Christian Tague, Zubayer Shams, Ruchi Jwalit Shah, Wanesa Wilczyńska, Hafiza Tooba Siddiqui, Muhammad Hanzalah Atif, Zarnab Tufail, Maria Qadri, Amidu Alhassan, Godfred Yawson Scott, Calvin R. Wei, Ankini Mukherjee, Mc Juan Muco Mugisha, Charles Bisimwa, Daniel Bulondo, Bonk Muhoza, Amos Kipkorir Langat

**Affiliations:** a Department of Research, Medical Research Circle (MedReC), Goma, DR Congo; b International Veterinary Vaccinology Network, The Roslin Institute University of Edinburgh, Edinburgh, United Kingdom; c Faculty of Sciences and Technologies, University of Kinshasa, Kinshasa, DR Congo; d Department of Medicine and Surgery, Jinnah Sindh Medical University, Karachi, Pakistan; e Brunel Medical School, Brunel University, Uxbridge, London, United Kingdom; f Department of Medicine, Nootan Medical College and Research Centre, Visnagar, Gujarat, India; g Department of Epidemiology and Tropical Medicine, Military Institute of Medicine—National Research Institute, Warsaw, Poland; h Department of Medicine and Surgery, Dow University of Health Sciences, Karachi, Pakistan; i Department of Public Health and Community Medicine, Gujranwala Medical College, Gujranwala, Pakistan; j College of Health and Allied Sciences, Department of Adult Health, University of Cape Coast, Cape Coast, Ghana; k Department of Medical Diagnostics, Kwame Nkrumah University of Science and Technology, Kumasi, Ghana; l Department of Research and Development, Shing Huei Group, Taipei, Taiwan; m Rampurhat Government Medical College and Hospital, Rampurhat, West Bengal, India; n Clinical Research Department, Rinda Ubuzima Research Organization, Kigali, Rwanda; o Pan African University for Basic Sciences Technology and Innovation, Nairobi, Kenya

**Keywords:** at-risk populations, children, Democratic Republic of Congo, malaria, malaria elimination, pregnant women

## Abstract

Malaria remains a significant public health challenge in the Democratic Republic of Congo (DR Congo), particularly among pregnant women and children under 5, who bear the highest burden of disease and mortality. Despite efforts to scale up control programs, the country faces numerous challenges, including limited healthcare access, inadequate funding, insecticide resistance, and gaps in surveillance. Vector control efforts are constrained by minimal government involvement and heavy reliance on international donors. Recent innovations, such as genomic surveillance, rapid diagnostic tests (RDTs), and mobile health (mHealth) solutions, offer new avenues for improving diagnosis, treatment, and data collection. Integrated approaches, such as incorporating malaria prevention into routine maternal and child healthcare, expanding access to vaccines like RTS,S and R21, and improving the distribution of long-lasting insecticidal nets (LLINs), are vital for sustaining control efforts. Community engagement and behavior change strategies are crucial to increasing uptake of preventive measures. Furthermore, climate change necessitates adaptable interventions to address shifting transmission patterns. Strengthening surveillance, training community health workers, and fostering public-private-philanthropic partnerships will enhance capacity for malaria control. Sustainable financing, policy reinforcement, and innovative tools are essential to prevent resurgence and protect vulnerable populations, ensuring progress toward malaria elimination in DR Congo. This review uniquely aims to address and consolidate evidence on pregnant women and young children in the DR Congo context, highlighting innovations and donor-dependence risks.”

## Introduction

Malaria remains a major public health concern in the Democratic Republic of the Congo (DR Congo), particularly affecting vulnerable populations such as pregnant women and young children. The country accounts for approximately 12% of global malaria cases and 13% of malaria-related deaths, reflecting a disproportionate share of the global burden^[1]^. This high impact is largely due to increased physiological vulnerability among these groups, coupled with limited access to preventive and curative services^[1,2]^. Children under 5 are at elevated risk of developing severe complications, including cerebral malaria and death, while malaria during pregnancy is associated with maternal anemia, low birth weight, and increased neonatal mortality^[3]^. Although interventions such as insecticide-treated nets (ITNs) and intermittent preventive treatment in pregnancy (IPTp) have been introduced, progress is hindered by weak healthcare infrastructure, antimalarial drug resistance, and logistical challenges in reaching remote populations^[4]^. Moreover, malaria recrudescence the recurrence of infection after initial treatment remains a persistent threat, especially in resource-limited settings like the DR Congo^[5]^. This resurgence is often linked to incomplete treatment adherence, resistance to frontline therapies, and inadequate post-treatment monitoring^[6]^. These challenges underscore the need for more robust surveillance systems and tailored, community-based interventions. A multifaceted approach is essential for malaria elimination. This includes strengthening health systems, promoting community awareness, ensuring continuous availability of effective antimalarial drugs, and scaling up preventive tools such as ITNs and vaccination. Integrating malaria control strategies into maternal and child health services can improve both coverage and health outcomes for high-risk groups^[7]^. This study explores strategies to support malaria elimination in the DR Congo, with a particular focus on protecting at-risk populations, especially pregnant women and children under 5, and minimizing the risk of malaria recrudescence.

## Methodology

This narrative review examines literature published between 2000 and 2025 related to malaria control and elimination in the DR Congo, with a focus on pregnant women and children under 5. A comprehensive search was conducted using PubMed, Google Scholar, and the World Health Organization repositories. The search strategy used for the databases included keywords like (“malaria” OR “malaria prevention and control” OR “malaria elimination” OR “eradication” OR “control”) AND (“Democratic Republic of Congo” OR “Democratic Republic of the Congo” OR DRC) AND (“pregnant women” OR “pregnancy” OR “maternal”) AND (“children” OR “child” OR “infant”) AND (“at-risk populations” OR “vulnerable populations” OR “risk groups”). The inclusion criteria comprised peer-reviewed and authoritative articles that were based on relevance, methodological rigor, contributed to understanding the epidemiology, interventions, and health system challenges in high-burden settings to capture policy and implementation perspectives. Any non-English or irrelevant papers were excluded. Possible duplicates were removed manually, and any discrepancies were resolved among the authors with mutual agreement.

## Main text

### Epidemiology of malaria in pregnant women and children under 5 in the DR Congo

Malaria is a fatal disease induced by Plasmodium species and spread by the bite of infected female Anopheles mosquitoes. Vectors globally contribute to about 700 000 deaths yearly, with malaria accounting for a notable contribution to morbidity and mortality in all ages^[8]^. The World Health Organization (WHO) reports that the number of malaria cases worldwide grew by 11 million from 2022 to 2023, totaling about 263 million cases, and resulted in about 597 000 deaths worldwide^[9]^. Despite being a preventable disease, malaria remains a major public health challenge. Global efforts have led to progress, with 44 countries and one territory certified malaria-free as of 2024. Of the 84 malaria-endemic countries, 25 now report fewer than 10 cases annually^[10]^. Sub-Saharan Africa is home to almost 80% of the world’s malaria burden, with only five countries (Nigeria, Mozambique, Uganda, and the DR Congo), which are responsible for almost half of all cases. The DR Congo contributes about 12% of the world’s malaria burden alone^[3,7]^.

**HIGHLIGHTS**

- The study investigates malaria prevalence, risk factors, and intervention strategies.
- Key findings indicate high infection rates among vulnerable populations, particularly pregnant women and children. Insecticide-treated nets (ITNs) and early diagnosis significantly reduce morbidity.
- Socioeconomic disparities and inadequate healthcare access hinder malaria control.
- The research underscores the importance of community engagement, enhanced vector control, and policy reinforcement.
- Strengthening surveillance, education, and healthcare infrastructure is essential to achieving malaria elimination goals.

**Figure 1.**
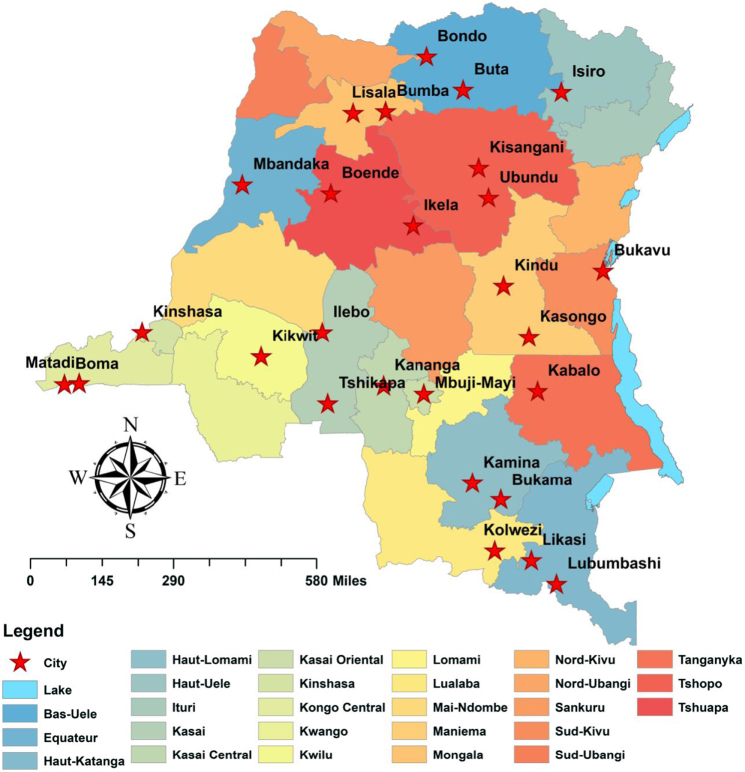
Geographic location of the study area in the DR Congo. This map illustrates the spatial distribution of areas where children aged 6–59 months tested positive for malaria by microscopy in different cities across the country.

The locations represented in Figure 1 give reference to where significant prevalence was recorded, highlighting areas at high risk of transmission. The purpose of this figure is to contextualize the study by showing the geographic disparities in malaria infection among young children.

Most malaria indicators have conventionally targeted children under 5. However, recent evidence indicates an increasing prevalence among older children and adolescents. While WHO and the Malaria Atlas Project (MAP) do not give precise age-specific estimates, a few studies report the malaria prevalence in children 2–10 as 36–38%^[11]^. Rural children have higher infection rates than urban children. Improved economic status, maternal education, and regular use of insecticide-treated nets (ITNs) are linked to lower malaria incidence in children^[12]^.

**Figure 2.**
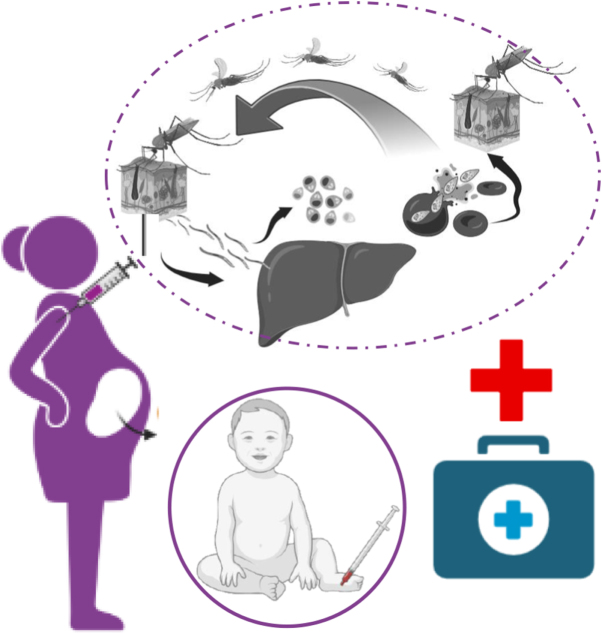
Malaria transmission patterns and increased vulnerability among pregnant women and children under 5 years of age.

In pregnant women, malaria infection tends to be asymptomatic, going unnoticed and potentially resulting in more severe complications of hypoglycemia, cerebral malaria, and pulmonary edema, complicating malaria-associated acute respiratory distress syndrome (MA-ARDS). Such complications can lead to maternal death and other pregnancy adversities such as prematurity, stillbirth, and miscarriage, all of which induce tremendous physical and emotional anguish^[13]^. Interestingly, Plasmodium falciparum infection in pregnancy can provoke protective antibodies that provide some immunity to subsequent pregnancies^[14]^. Figure 2 shows the principal mechanisms of malaria transmission, with the participation of the female Anopheles mosquito in the spread of the Plasmodium parasite and biological as well as epidemiological effects on risk populations. It exposes pregnant women to problems, including maternal anemia and placental malaria, as well as poor pregnancy outcomes. Children of less than 5 years of age are highly susceptible because their immune systems are not developed to a high level and, therefore, are the target of prevention strategies. This graphical illustration demonstrates the dire need to use an intervention targeting vulnerable populations in order to decrease the burden of the disease in these communities.

Malaria is the predominant cause of disease and death among children under age 5 in the DRC, with an estimated mortality rate of 40%. This morbidity can be lessened through ongoing government and NGO interventions, including ITN distribution, indoor residual spraying, and access to antimalarial drugs^[1,15]^.

### *Current approaches to fighting malaria in the DR Congo* (Fig. 3)

#### Intermittent preventive treatment in pregnancy (IPTp)

Pregnant women are especially at risk of malaria, with serious implications for both mother and child^[16]^. WHO advises intermittent preventive treatment with sulfadoxine-pyrimethamine (IPTp-SP) at antenatal consultations in areas of moderate to high transmission^[16]^. IPTp-SP delivery through the community has been demonstrated to enhance uptake without compromising attendance at antenatal clinics^[17,18]^. Nevertheless, access is still limited in parts of the DR Congo, where only 22–24% of pregnant women are getting three doses, as recommended^[19]^.

**Figure 3.**
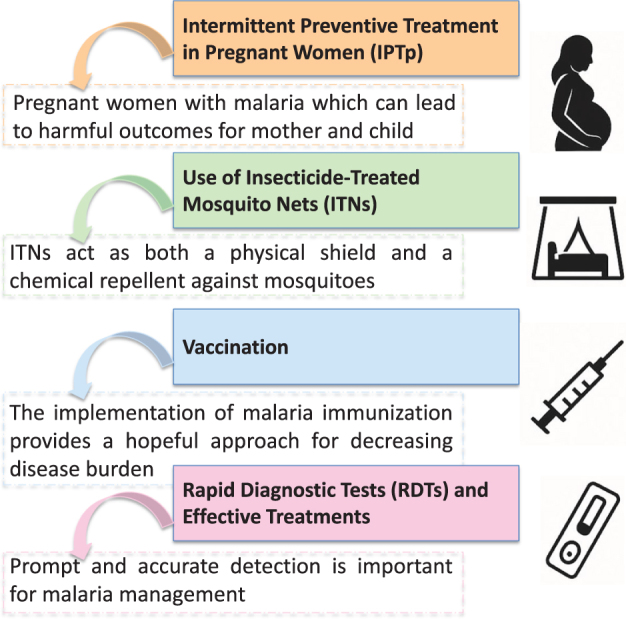
Current malaria control strategies in the DR Congo at a schematic view. This is the summary of the major interventions of public health that have been used in alleviating malaria burden to the nation. It involves intermittent preventive treatment during pregnancy (IPTp-SP), use of insecticide-treated nets (ITNs), *Plasmodium falciparum* immunization campaigns in addition to getting access to rapid diagnostic tests (RDTs) and effective antimalarial treatments. Another aspect of these blended strategies is to focus on the weakest population or pregnant women and children younger than 5 years; and also, to intensify surveillance, prevention, and care where the transmission is high.

#### Insecticide-treated nets (ITNs)

ITNs offer a physical and chemical barrier against mosquitoes and have been instrumental in reducing malaria incidence in most parts of the world^[20]^. In the DR Congo, improper usage of ITNs has also been associated with anemia during pregnancy and low birth weight. Although mass distribution campaigns have been extensive; steady use is still a problem owing to cultural beliefs, durability of nets, and low awareness^[21]^.

#### Vaccination

The malaria vaccine is a promising intervention for the control of disease burden. The RTS,S/AS01 vaccine has been recommended by the WHO and implemented in a number of African nations^[22]^. Furthermore, the R21/Matrix-M vaccine, which was produced by the University of Oxford and Serum Institute of India, has shown efficacy in clinical trials and was prequalified by the WHO in 2023^[23]^. These vaccines work by targeting the *Plasmodium falciparum* circumsporozoite protein for blocking liver-stage infection^[21]^. Implementation difficulties involve shortages in supply, cold chain needs, and incorporation into current health systems^[22,24]^. A recent pattern in the cost-effectiveness model of the R21/Matrix-M vaccine in sub-Saharan Africa indicates that in areas of constantly high transmission rates, the cost per disability-adjusted life year (DALY) prevented may be as low as US$30–34, making it competitive with other preventive tools and economically feasible^[25]^.

#### Rapid diagnostic tests (RDTs) and effective treatment

Early diagnosis and accurate diagnosis are vital for malaria control. RDTs give immediate results, allowing timely treatment, particularly in rural areas^[10]^. The DR Congo has made efforts to enhance access to RDTs, although problems with supply chain breakdowns and variable test quality remain^[26]^. Artemisinin-based combination therapies (ACTs) are still the first-line treatment; however, increasing resistance to artemisinin in certain areas of Africa is a danger^[19]^. Ongoing surveillance of resistance and research into new drugs are crucial^[8,14,27]^.

A successful malaria response in the DR Congo needs to incorporate preventive measures, vaccination, prompt diagnosis, and treatment. Challenges such as resistance, irregular use of ITNs, and weak healthcare infrastructure need to be overcome in order to ensure sustainable gains^[1]^.

### Challenges and implementation gaps of control programs

The DR Congo experiences several challenges in the implementation of malaria control programmes across the country. Vector control is hampered by an absence of holistic data on the mosquito species, pattern of transmission, and insecticide resistance. There is only one national publication that currently informs such efforts^[28]^.

Limited government expenditure limits access to healthcare, particularly in rural areas. The nation depends on international donors, and private firms (primarily in mining) manage localized vector control^[29]^. Resistance of Anopheles gambiae to DDT and permethrin was documented in 2009, highlighting the need to manage resistance urgently. Although DDT usage is controlled by the Stockholm Convention, the vector is still susceptible to deltamethrin, and organophosphates remain effective^[30,31]^.

National programmes for malaria control should place greater emphasis on specific vector control interventions and improved social and behavior communication to achieve regular IPTp administration, particularly among younger pregnant women^[32]^. Economic costs of uncomplicated malaria cases emphasize the necessity to truncate pre-hospital delays and cover social inequalities through better national control programmes^[33]^.

During a 2014 campaign, roughly 3.5 million nets were distributed using fixed-site and door-to-door strategies. Fixed-site delivery was less expensive and had superior coverage. Maintenance of the gains is essential^[34]^. Community mobilization through health seminars and enhanced rural health center supply chains are also essential. Better data collection through regional partnerships and local surveillance systems is required.

Global coordination, such as public-private-philanthropic collaboration and enhanced cooperation with nations like India, may be used to support research funding, institute sustained vector management practices, and develop local capacity^[35]^. Coordination between urban and rural programmes is needed to provide equitable distribution of resources and sustained impact.

### Strengthening programmes to secure elimination gains

Inclusion of malaria prevention in maternal and child health is key to maintaining elimination gains. Routine administration of antimalarial drugs such as sulfadoxine-pyrimethamine (SP) to pregnant women in endemic regions, as advocated by WHO^[3]^, is important. ITNs and LLINs continue to be important for protection^[31]^. Their distribution needs to be scaled up to achieve complete coverage in pregnant women.

Correspondingly, immunization activities should aim to ensure the availability of RTS,S and R21 vaccines in primary health facilities, especially in high-burden districts. Both vaccines have been validated by WHO for safety and efficacy in children^[6]^.

New surveillance technologies, including genomic surveillance, allow public health experts to track drug resistance and patterns of transmission. This facilitates more targeted, evidence-based interventions^[31]^. RDTs enhance diagnostic sensitivity and availability where laboratory capacity is weak^[36]^.

Recent developments in vector control are genetically modified mosquitoes (GMMs) and biopesticides, which are safer substitutes for chemical insecticides. Technology in the form of mobile health (mHealth), geographic information systems (GIS), and electronic health records (EHRs) has also improved data collection and reporting^[28]^. There is a need for an expert workforce. Community Health Workers (CHWs) must be trained in diagnosis, treatment, and patient education. Outreach Training and Supportive Supervision (OTSS) has been shown to be effective in enhancing health worker performance^[37]^. More research must be undertaken in order to create climate-resilient malaria interventions. Involving the private sector with funding and awareness programmes can enhance efforts. Public-private-philanthropic collaborations and assistance from the WHO, the Global Fund, and the President’s Malaria Initiative (PMI) are essential for large-scale scaling of control measures.

### *Preventing resurgence and protecting at-risk populations* (Fig. 4)

Malaria continues to be a severe health concern in the DR Congo, particularly among pregnant women and children under 5 years of age. In 2022, it had over 27 million reported cases, with almost half these cases involving children under 5, who represented approximately 70% of malaria fatalities^[26]^.

**Figure 4.**
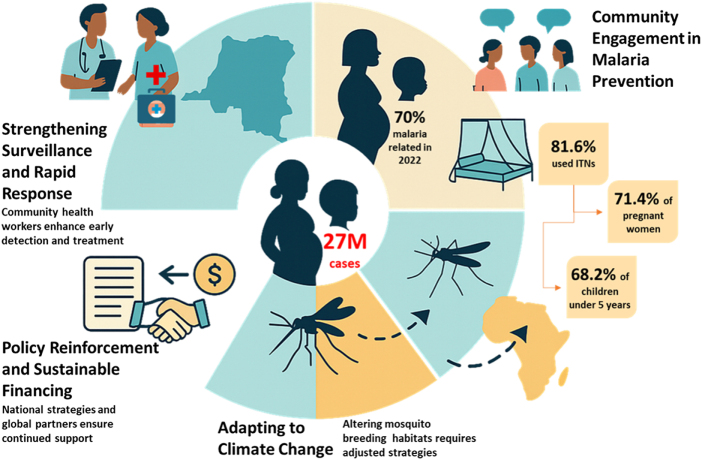
Visual illustration of integrated malaria prevention approaches to DR Congo with emphasis on the most vulnerable segment of the population, including pregnant women and children under the age of 5. The illustration displays the pillars of a sustainable strategy community surveillance, community engagement, quality use of insecticide-treated nets, policy strengthening at the national level, sustainable sourcing of funds and adaptation to the climate changes impact on the transmission process. It also emphasizes the need to have synergy between local and international players to alleviate resurgence to minimize the death caused by malaria infection.

#### Surveillance and rapid response

A robust surveillance system is indispensable for early detection and outbreak containment. CHWs are pivotal for diagnosing and treating malaria in peripheral settings, improving early intervention, and decreasing transmission^[38]^. Fusing local data with national systems facilitates real-time responses.

#### Community engagement in malaria prevention

Community engagement is essential for effective malaria control, especially among pregnant women and young children. In the DR Congo, insecticide-treated mosquito nets (ITNs) are a primary preventive measure. A study revealed that while 81.6% of households possessed an ITN, only 71.4% of pregnant women and 68.2% of children under 5 used them the preceding night. Factors such as positive attitudes toward ITNs and confidence in their use significantly increased usage rates. These findings highlight the need for behavior change interventions to boost ITN utilization among vulnerable groups^[39]^. Educating communities fosters ownership of malaria control strategies, enhancing adherence to preventive measures.

#### Policy reinforcement and sustainable financing

Strong policies and adequate funding are necessary for malaria elimination. The DR Congo’s National Malaria Control Program (NMCP), in collaboration with global partners, has introduced a National Malaria Advocacy and Resource Mobilization Strategy as part of the Malaria Strategic Plan 2024–2028^[40]^. Sustainable financing ensures continued access to insecticide-treated mosquito nets, effective treatments, and vaccination programmes.

#### Adapting to climate change and emerging trends

Climate change is influencing malaria transmission by altering mosquito breeding habitats. Adjusting malaria control strategies to changing transmission patterns is essential. Preventive measures, such as reducing stagnant water and improving sanitation, help control mosquito populations. Continuous monitoring enables health authorities to modify interventions as required^[41]^. To add on, projections of climate-driven shifts in malaria suitability zones suggest that areas near the Albertine Rift (DRC-Uganda border) could become increasingly significant for higher transmission risk under suitable warm conditions, augmenting vulnerable patients’ susceptibility to malaria^[42]^.

### Recommendations

To strengthen malaria control, programmes should be more deeply integrated into primary healthcare systems, supported by improved health education and community engagement^[43]^. Technological advances, including real-time data collection, geospatial mapping, and enhanced surveillance, should be leveraged to guide targeted interventions^[44]^. Drug and insecticide resistance must be addressed through the development of new therapies, insecticide rotation, and innovative solutions like drone-assisted larval source management^[45,46]^. Finally, collaboration among governments, NGOs, and communities, backed by sustainable financing and stronger governance, is essential for the success and longevity of malaria elimination efforts in DR Congo.

## Conclusion

Malaria continues to pose a serious health burden in the Democratic Republic of Congo, disproportionately affecting pregnant women and young children. Despite decades of control efforts, the country continues to experience high transmission rates, driven by a combination of biological, environmental, and systemic factors. Resistance to antimalarial drugs and insecticides, gaps in prevention coverage, and limited health infrastructure have all contributed to the persistence of the disease. Recent innovations, including new vaccines and digital health technologies, offer hope for improved outcomes. However, their benefits have yet to be fully realized due to logistical and operational constraints. This review highlights the complexity of malaria control in high-burden settings and underscores the need for sustained attention. Understanding the interaction between disease dynamics, population vulnerability, and health system capacity is crucial for effective disease management. Without continuous adaptation and strong national commitment, gains achieved over recent years risk being lost, and malaria may continue to undermine health and development.

## Data Availability

Not applicable.
